# Ferritin above 100 mcg/L could rule out colon cancer, but not gastric or rectal cancer in patients with involuntary weight loss

**DOI:** 10.1186/1471-230X-12-86

**Published:** 2012-07-09

**Authors:** Cristian Baicus, Simona Caraiola, Mihai Rimbas, Ruxandra Patrascu, Anda Baicus

**Affiliations:** 1 Department of Internal Medicine, Colentina University Hospital, Soseaua Stefan cel Mare 19-21, sector 2, Bucharest, 020125, Romania; 2 Department of Gastroenterology, Colentina University Hospital, Soseaua Stefan cel Mare 19-21, sector 2, Bucharest, 020125, Romania; 3 Clinical Research Unit RECIF (Réseau d’ Epidémiologie Clinique International Francophone), Bucharest, Romania; 4 I. Cantacuzino” National Institute of Research and Development in Microbiology-Immunology, Splaiul Independentei 103, sector 5, Bucharest, 050096, Romania; 5 University of Medicine and Pharmacy “Carol Davila”, Bucharest, Romania

**Keywords:** Area under the curve (AUC), Gastrointestinal cancer, Colon cancer, Ferritin, Involuntary weight loss, Sensitivity and specificity

## Abstract

**Background:**

A tenth of patients with involuntary weight loss (IWL) have gastrointestinal cancer. Ferritin is the first parameter to be modified during the process leading to iron deficiency anaemia, therefore it should be the most sensitive. The aim of this study was to assess the ability of ferritin to rule out gastrointestinal cancer in patients with involuntary weight loss.

**Methods:**

All consecutive patients with IWL admitted in a secondary care university hospital were prospectively studied. Ferritin, haemoglobin with erythrocyte indices and serum iron were recorded for all patients. The reference standard was bidirectional endoscopy and/or 6 months follow-up.

**Results:**

290 patients were included, a quarter had cancer, of which 22 (7.6%) had gastrointestinal cancer (8 gastric cancer, 1 ileum cancer, 13 colorectal cancer). Ferritin had the best area under the curve (AUC), both for gastrointestinal cancer (0.746, CI: 0.691-0.794), and colorectal cancer (0.765, CI: 0.713-0.813), compared to the other parameters of iron deficiency. In the diagnosis of colorectal cancer, ferritin with a cut-off value of 100 mcg/L had a sensitivity of 93% (CI: 69-100%), and negative likelihood ratio of 0.13, with a negative predictive value of 99% (96-100%), while for gastrointestinal cancer, the sensitivity was lower (89%, CI: 67-95%), with a negative likelihood ratio of 0.24. There were three false negative patients, two with gastric cancer, and one with rectal cancer.

**Conclusion:**

In patients with involuntary weight loss, a ferritin above 100mcg/L could rule out colon cancer, but not gastric or rectal cancer.

## Background

Involuntary weight loss (IWL) is an important health problem, as 3-5% of the patients in internal medicine departments are admitted for this [1-3], and among them, a quarter have cancer [4], part of them having gastrointestinal cancer. For this reason, gastrointestinal endoscopy is recommended in the diagnosis of IWL, after the initial investigations consisting in history and physical examination, chest radiography, abdominal ultrasonography and standard laboratory tests [2,5].

As it is known, iron deficiency anaemia appears in gastrointestinal cancer due to occult blood loss. However, anaemia as a diagnostic test for gastrointestinal cancer is not sensitive, because a high proportion of patients with gastrointestinal cancer (50%) do not have anaemia at the moment of tumour diagnosis [6-8].

In the succession of events that leads to iron deficiency anaemia, ferritin diminution appears early, immediately after the waning of the iron stores in the bone marrow, and before occurrence of all the other changes in iron deficiency anaemia: decrease in transferrin saturation, serum iron, red cell volume and haemoglobin [9], and this allows us to hope that ferritin would have a higher sensitivity for the diagnosis of gastrointestinal cancer. In fact, ferritinaemia is the test with the highest accuracy in the diagnosis of iron deficiency anaemia compared to the above-mentioned parameters [10-12].

Serum ferritin was assessed in a few, retrospective studies for the diagnosis of gastrointestinal cancer, and the results showed that it has no value as a screening test [13], probably because it lowers only when the tumour has grown enough [14], condition that might be satisfied in the case of IWL [15]. In one study, 10% of the 143 patients having a ferritin less than 50 mcg/L had gastrointestinal cancer [16], in two patients without anaemia, a low ferritin (<18 mcg/L) lead to the discovery of colon cancer [17], and in 414 patients referred for colonoscopy because of anaemia, a ferritin higher than 100 mcg/L excluded colorectal cancer [18]. However, in a retrospective study on 359 consecutive elderly inpatients referred to colonoscopy because of symptoms suggesting colorectal cancer, ferritin was not useful in the diagnosis [19].

To the date, no studies were done in patients with IWL.

We therefore conducted a diagnostic study to determine whether ferritin higher than 100 mcg/L is sensitive enough to exclude gastrointestinal cancer in patients with IWL.

## Methods

### Setting and patients

We prospectively studied adult patients referred for IWL (without evident origin after clinical assessment), admitted as inpatients or referred to the day hospital in the Departments of Internal Medicine of a secondary care university hospital. All consecutive patients, 18 years of age or more, were included if they fulfilled one of the following criteria: 1) documented IWL of at least 5% of body weight within the last 6 months, or 2) declaration of a “very much” or “much” concerning the amount of weight loss, on a Lickert scale with five levels (“very much”, “much”, “average”, “little” and “not at all”). The second criterion was applied only for the selection of patients for whom there was no baseline weight documentation, and the amount of weight loss could not be computed in order to fulfil the first criterion; for these patients, the existence of the weight loss had to be confirmed by a relative, or by changes in clothes or belt size. The patients with voluntary weight loss or with a known malignancy were not included in the study.

The study was conducted according to the Declaration of Helsinki, the protocol was approved by the ethics committee of Colentina University Hospital and all patients agreed to participate in the study and signed the informed consent before enrolling.

### Study design

The investigative work-up was not standardized; it was decided by every participating physician, depending on clinical and biological diagnostic clues - therefore only patients in whom gastrointestinal cancer was suspected had upper endoscopy and/or colonoscopy. In order to avoid misclassification concerning the final diagnosis (gastrointestinal cancer or not) for patients who did not have endoscopic studies, after leaving the hospital all the patients were followed up for six months, verifying the final diagnosis, survival, state of health and further weight change.

All patients had blood collected at admission. Because ferritin was measured especially for this study, blood was centrifuged and the serum was frozen at −70°C; ferritin level was determined later in another laboratory, therefore the physicians and the endoscopists were blinded to the results. As one of the objectives of the research project was the validation of several simple clinical and biological parameters, found as diagnostic for cancer in IWL patients in three previous studies [20-22], all patients had a complete blood cell count (including MCV and RDW) and determination of blood erythrocyte sedimentation rate (ESR), serum C reactive protein (CRP), iron, albumin, alkaline phosphatase (ALP), alanin aminotranspherase (ALAT) and lactate dehydrogenase (LDH) levels, determined in the hospital laboratory. All variables were first recorded by every physician in a preformed questionnaire, and then registered by one of the authors into the database.

### Laboratory procedures

The RDW, haemoglobin level and MCV were determined using the Sysmex XT 1800i counter (Sysmex Corporation, Kobe, Japan). Ferritin was measured with Chemwell 2910 analyzer (Awareness Technology, Palm City, Florida, USA), while serum albumin, iron, ALP, ALAT, LDH and CRP levels were measured using the Cobas 6000 Modular P 800 analyzer (Roche Diagnostics, Rotkreuz, Switzerland). ESR was measured by Westergreen method. Throughout the study, the quality of results was validated by regular internal quality control procedures and participation in an external quality assessment scheme.

### Statistical analysis

The outcomes were gastrointestinal cancer/colorectal cancer as a cause of IWL, while the predictor variable was ferritin.

Results were expressed as frequencies for categorical variables, and median and extremes for non normal continuous variables. Stata 11 (StataCorp, College Station, Texas, USA) was used for the database construction and data analysis (area under the curve calculation with confidence intervals, and logistic regression). Hypothesis testing was 2-tailed, with *P* < .05 considered statistically significant. EBM calculator 1.0 (http://www.cebm.utoronto.ca) was used for the calculations of sensitivity, specificity, predictive values and likelihood ratios.

## Results

### Patients: inclusion, work-up and follow-up

**Figure 1:**
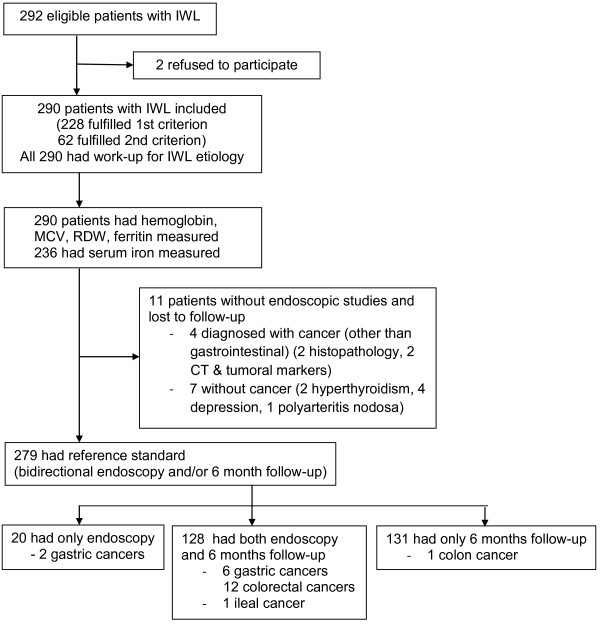
Flow diagram of the study.

292 consecutive patients with IWL were admitted from January 2009 to September 2010 (flow diagram presented in Figure 1). Two patients refused to participate, therefore 290 patients were included in the present study – 228 fulfilled the first inclusion criterion (baseline weight was known, so the amount of weight loss could be computed), while the other 62 fulfilled the second criterion.

All admitted patients had hospital work-up and determination of ferritin. 279 (96%) patients had the reference standard (bidirectional endoscopy and/or 6 months follow-up of which 140 patients had bidirectional endoscopy), while 11 patients did not have endoscopy and were lost to follow-up after leaving the hospital: four were diagnosed with cancer, other than gastrointestinal (two by histopathology, and two by a combination of computed tomography and elevated serum tumour markers), while the other seven patients left the hospital without a cancer diagnosis (two were diagnosed with hyperthyroidism, four with depression, and 1 with polyartheritis nodosa). The eight patients diagnosed with gastric cancer had only upper endoscopy, considering this cancer as the cause of their weight loss, therefore the colonoscopy was not performed; of these patients, 6 were followed-up for six months.

The characteristics of the patients, together with the results of the haematological and inflammation tests (including ferritin) are provided in Table 1.

**Table 1:** Patients’ characteristics

**Characteristic**	**Patients without cancer**	**Patients with gastrointestinal cancer**	**Patients with other cancers**
Age (yr)	67 (22, 94)	70 (55, 82)	66 (44, 93)
Male sex	104 (48%)	12 (54%)	30 (58%)
RDW (%)	14.5 (10.3, 26.7)	14.8 (12.3, 20.4)	15.1 (11.5, 20.8)
Haemoglobin (g/dL)	12.6 (6.14, 17.4)	11.3 (7.9, 15.1)	11.8 (5.42, 17.7)
MCV (fL)	88 (62, 124)	84.5 (64, 98)	88.2 (72, 102)
Serum iron (mcg/dL)	54 (10, 197)	35.5 (10, 118)	35 (4, 162)
Ferritin (mcg/L)	99.5 (3, 2000)	26.5 (10, 500)	226 (15, 1105)
ESR (mm/h)	28 (2, 140)	44.5 (3, 140)	58 (11, 140)
CRP (mg/L)	5 (0.51, 328)	22 (1.6, 125)	46 (1, 550)
ALAT (u/L)	18 (6, 325)	18 (10, 71)	26 (10, 143)
Total number	216	22	52

### Outcome

22 patients were diagnosed with gastrointestinal cancers (7.6%) (8 gastric cancers, 1 ileum cancer, 10 colonic cancers and 3 rectal cancers). 148 patients (51%) had upper endoscopy, and 140 patients (48%) had colonoscopy performed.

As seen in Table 2, ferritin had the best area under the curve (AUC), both for gastrointestinal cancer (0.746, CI: 0.691, 0.794), and colorectal cancer (0.765, CI: 0.713, 0.813), while haemoglobin, mean red cell volume (MCV) and serum iron performed worse; red cell distribution width (RDW) was not useful.

**Table 2:** Areas under the curve for ferritin, MCV, serum iron, haemoglobin and RDW in the diagnosis of gastrointestinal cancer

	**Gastrointestinal cancer**	**Colorectal cancer**
	**AUC (95% CI)**	**AUC (95% CI)**
Ferritin	0.746 (0.691-0.794)	0.765 (0.713-0.813)
MCV	0.627 (0.568-0.684)	0.669 (0.612-0.725)
Serum iron	0.636 (0.571-0.697)	0.650 (0.584-0.710)
Haemoglobin	0.590 (0.530-0.646)	0.552 (0.491-0.609)
RDW	0.541 (0.476-0.602)	0.463 (0.401-0.528)

For a cut-off value of 100 mcg/L, ferritin had a sensitivity of 93% (CI: 69-100%) for colorectal cancer, with a negative likelihood ratio of 0.13, and a negative predictive value of 99% (CI: 96-100%), for a prevalence of 5% (Table 3). The parameters were lower when gastrointestinal cancer was considered instead of colorectal cancer. In the same table there are presented the parameters for a ferritin cut-off value of 50 mcg/L and anaemia as diagnostic tests for colorectal and gastrointestinal cancer (anaemia was defined using the World Health Organization definition of haemoglobin less than 13 g/dL for men, and less than 12 g/dL for women) [23].

**Table 3:** Ferritin with cut-off values of 100 mcg/L and 50 mcg/L, and anaemia in the diagnosis of gastrointestinal cancers (in paranthesis are 95% CI)

		**Gastrointestinal cancer**	**Colorectal cancer**
Ferritin (cut-off 100 mcg/L)	Sensitivity (%)	86 (67–95)	93 (69–100)
	Specificity (%)	57 (51–62)	56 (50–62)
	Positive predictive value (%)	14 (9–21)	10 (6–16)
	Negative predictive value (%)	98 (94–100)	99 (96–100)
	Positive likelihood ratio	2	2.1
	Negative likelihood ratio	0.24	0.13
	Sensitivity (%)	59 (39–77)	64 (39–84)
	Specificity (%)	76 (71–81)	75 (70–80)
Ferritin (cut-off 50 mcg/L)	Positive predictive value (%)	17 (10–27)	12(6–20)
	Negative predictive value (%)	96 (92–98)	98 (95–99)
	Positive likelihood ratio	2.5	2.6
	Negative likelihood ratio	0.54	0.47
	Sensitivity (%)	68 (47–84)	64 (39–84)
	Specificity (%)	51 (45–57)	50 (44–56)
Anaemia	Positive predictive value (%)	10 (6–16)	6 (3–11)
	Negative predictive value (%)	95 (90–98)	97 (92–99)
	Positive likelihood ratio	1.4	1.3
	Negative likelihood ratio	0.63	0.71

In a multivariable model where the haematological parameters, together with age and sex were entered, only ferritin was associated with gastrointestinal and colorectal cancer (Table 4): after controlling for age, sex, haemoglobin, serum iron and red cell indices, patients with ferritin <100 mcg/L were 7 times more likely to have gastrointestinal cancer and 11 times more likely to have colorectal cancer. The results were not changed when ESR, CRP and ALAT were introduced into the model in order to evaluate the influence of inflammation and alcohol consumption (Additional file 1). In bivariate analysis, we found that ferritin was statistically associated to serum inflammatory markers and alcohol consumption, although the association was weak (Kendall’s r = 0.252 with ESR, 0.279 with CRP and 0.235 with ALAT, p < 0.001).

**Table 4:** Haematological parameters, together with age and sex, as predictors of colorectal/gastrointestinal cancer in patients with IWL – logistic regression

	**Variable**	**Odds ratio**	**95% CI**	***P*value**
Colorectal	sex (male)	1.18	0.26, 5.36	0.824
cancer	age (years)	1.08	0.99, 1.17	0.053
(14 patients)	hemoglobin (g/dl)	0.84	0.53, 1.34	0.473
	**MCV (femtoliters)**	0.87	0.76, 0.99	**0.049**
	serum iron (mcg/dl)	0.99	0.96, 1.02	0.601
	RDW (%)	0.70	0.43, 1.13	0.148
	**ferritin < 100 mcg/L**	**11.57**	**1.34, 99.21**	**0.026**
Gastrointestinal	sex (male)	1.54	0.51, 4.68	0.443
cancer	age (years)	1.05	0.99, 1.10	0.060
(22 patients)	hemoglobin (g/dl)	0.86	0.64, 1.51	0.310
	MCV (femtoliters)	0.97	0.89, 0.05	0.441
	serum iron (mcg/dl)	0.99	0.97, 1.01	0.392
	RDW (%)	0.98	0.74, 1.31	0.906
	**ferritin < 100 mcg/L**	**6.96**	**1.84, 26.27**	**0.004**

Ferritin was statistically associated with gastrointestinal/colorectal cancer only in patients with anaemia, and areas under the curve performed better in these patients than in the whole sample (AUC = 0.799, CI: 0.688-0.910 for gastrointestinal cancer, and 0.839, CI: 0.765-0.913 for colorectal cancer) (Table 5).

**Table 5:** Ferritin and gastrointestinal/colorectal cancer in patients with and without anemia

		**Gastrointestinal cancer**		**Colorectal cancer**	
			**AUC (95%CI)**		**AUC (95%CI)**
Anemia, present (145 patients, 15/9 with gastrointestinal/ colorectal cancer)	Ferritin	P < 0.001^*^		P < 0.001^*^	
	(100mcg/L)				
	Ferritin	P < 0.001^*^		P = 0.005^*^	
	Ferritin (mcg/L)	**P = 0.001**^†^	**0.799**	**P < 0.001**^†^	**0.839**
	(50 mcg/L)				
			**(0.713-0.813)**		**(0.765-0.913)**
Anemia, absent (142 patients, 7/5 with gastrointestinal/ colorectal cancer)	Ferritin (100mcg/L)	P = 0.125^*^		P = 0.375^*^	
					
	Ferritin	P = 0.196^*^		P = 0.154^*^	
	(50 mcg/L)				
	Ferritin (mcg/L)	P = 0.536 ^†^	0.663	P = 1.000^†^	0.635
			(0.468-0.859)		(0.391-0.880)

^*^ Fisher’s exact test.

^†^ Mann–Whitney *U* test.

## Discussion

Endoscopic investigations (both upper and lower) are recommended immediately after history, physical examination, chest radiography, abdominal ultrasonography and standard laboratory tests in patients with IWL [2,5], because gastrointestinal cancer is always feared, although its frequency is about 10% in patients with IWL [5,15,20]. In our study on 290 patients, 148 patients (51%) were investigated using upper endoscopy, and 140 patients (48%) had colonoscopy, while the frequency of gastric and colorectal cancer was only 2.8% and 4.5%, respectively.

Generally, the sensitivity of anaemia for the diagnosis of gastrointestinal cancer is only about 50% [6-8], but theoretically it should be more sensitive in IWL, as more advanced tumours are usually diagnosed in these patients [15]. On the other hand, ferritin should be more sensitive in the diagnosis of iron deficiency states, therefore in the diagnosis of gastrointestinal cancer as the cause of weight loss.

Ferritin was assessed in a few studies for the diagnosis of gastrointestinal cancer, and revealed that 10% of 143 patients with a ferritin less than 50 mcg/L had gastrointestinal cancer [16]; in two patients without anaemia, a low ferritin (<18 mcg/L) actually lead to the discovery of colon cancer [17]; and in 414 patients referred for colonoscopy because of anaemia, a ferritin higher than 100 mcg/L excluded colorectal cancer [18]. However, in a retrospective study on 359 consecutive elderly inpatients referred to colonoscopy because of symptoms suggesting colorectal cancer, ferritin was not useful in the diagnosis [19].

In the current study, we examined the usefulness of ferritin in the diagnosis of gastrointestinal/colorectal cancer in patients with IWL, and we found that ferritin was a better test than serum iron, MCV, haemoglobin, and RDW, as it had the biggest AUC, and it was the only test associated with gastrointestinal and colorectal cancer in multivariable analysis.

Ferritin ≥ 100 mcg/L could exclude colon cancer, because it had good sensitivity and negative likelihood ratio, and a very high negative predictive value. The main weakness of the study is the low prevalence of colon cancer in this series of patients with IWL, possibly overestimating the negative predictive value of ferritin ≥ 100 mcg/L, and leading to a relatively large confidence interval for the sensitivity.

The cut-off value of 50 mcg/L for ferritin is useless, because the test looses sensitivity, without a decisive gain in specificity, while anaemia is, as expected, a little more sensitive for gastrointestinal cancer in these patients with IWL than in other patients, but not sensitive enough to rule out the disease.

Ferritin < 100 mcg/L was less sensitive for gastrointestinal cancer, because of two false negative gastric cancers, the third false negative being a rectal cancer. Upon this results, one could speculate that ferritin might be very sensitive only for colon cancer, whose main feature is iron deficiency anaemia, and not for the extremities of the digestive tract (stomach and rectum), which are, anyway, easier to explore by upper endoscopy and rectoscopy (Additional file 2).

Ferritin as a diagnostic test performed much better than haemoglobin both in terms of AUC and sensitivity. Using ferritin in place of haemoglobin, 6 additional patients without anaemia were diagnosed with cancer (1 with caecum cancer, 2 with stomach cancer, 2 with rectal cancer, and one with sigmoid cancer) (Table 6).

**Table 6:** Ferritin, ESR, CRP, ALAT and alcoholism^*^in patients with gastrointestinal cancer

**Localisation of cancer**	**nr**	**Ferritin**	**Anaemia**	**ESR**	**PCR**	**ALAT**	**Alcoholism**
CAECUM	1	38	**NO**	16	5.7	12.7	NO
COLON	1	71	YES	**101**	**77.4**	13	NO
(ascending,	2	82	YES	**140**	**125**	30	NO
transverse,	3	26	YES	**57**	**25.6**	21.7	NO
descending)	4	10	YES	**60**	1.7	12	NO
	5	20	YES	**71**	**53**	12	NO
	6	14	YES	**44**	**29.6**	14.6	NO
	7	17	YES	**45**	**22**	**34**	**YES**
STOMACH	1	16	**NO**	10	1.59	15	NO
	2	87	YES	**53**	**110**	**47**	**YES**
	3	12	YES	**44**	**40**	24	NO
	4	**108**	YES	29	**26.5**	**71**	**YES**
	5	15	YES	24	**18.7**	24	NO
	6	**500**	YES	**90**	**51**	30	NO
	7	86	**NO**	36	**21.7**	18	NO
	8	11	YES	40	**20.7**	10	NO
ILEUM	1	58	YES	**49**	**35**		NO
RECTUM	1	27	**NO**	**51**	**36.5**	13	NO
	2	**322**	**NO**	**56**	**27**	31	NO
	3	18	**NO**	3	7.3	19	NO
SIGMOID	1	23	YES	16	16	10	NO
	2	83	**NO**	18	3	10.4	NO

^*^ alcoholism as discharge diagnosis.

The fact that ferritin was not associated with gastrointestinal/colon cancer in patients without anaemia was unexpected. This finding could be explained, in our opinion, by the low statistical power (only 7 and 5 patients without anaemia had gastrointestinal and colon cancer, respectively), and by the fact that, in this population with IWL, ferritin was more homogenous in patients without anaemia, who generally did not have an organic disease, while being more frequently elevated in patients with anaemia of chronic disease, or low in patients with iron deficiency anaemia due to gastrointestinal cancer [24].

As it is known, ferritin increases in inflammation and alcohol consumption, which means that, if adjusted for inflammation, this test could have become even better as sensitivity (with fewer false negatives), and therefore for ruling out the disease. However, there are no guidelines concerning this adjustment: one source suggests that inflammation augments the serum ferritin threefold, and therefore its value should be divided by three [9], without any research study as reference, while a meta-analysis showed that inflammation augments the value of ferritin with about 40% [25], but it is based mainly on studies on children with acute inflammation. Nowhere is stated from what level of inflammation one should adjust, and if adjustment should be the same for a CRP of 10 as for a CRP of 80 mg/L. This eventual adjustment could have changed the classification of only 3 patients with ferritin higher than 100mcg/L who tested false negatives (two with stomach cancer and one with rectal cancer (Table 6), but it is probably that the patient with stomach cancer and ferritin of 500 mcg/L would have been a false negative regardless of any such adjustment, because even if divided by three, the value still would have been higher than 100 mcg/L.

Because IWL can have multiple causes, it is difficult, expansive and frequently useless to settle a standardized investigation protocol [2]. We decided to let the investigators decide the diagnostic approach in every patient, depending on clinical and biological diagnostic clues. In order to avoid misclassification concerning the final diagnosis (gastrointestinal cancer or not) for patients who did not have both upper and lower digestive endoscopy, after leaving the hospital the patients were followed up for six months, therefore our diagnostic study had a valuable reference standard. Only 11 (3.8%) patients did not have endoscopy and were lost to follow-up after leaving the hospital, of which only 4 had not a certain cause for their weight loss. All patients had ferritin measured.

## Conclusions

Our study demonstrates that, in patients with involuntary weight loss, a ferritin above 100mcg/L could rule out colon cancer, but not gastric or rectal cancer.

## Competing interests

The authors declare that they have no competing interests.

## Authors’ contributions

CB conceived the study and performed data analysis. CB and SC wrote the protocol and introduced data into the database. CB and MR wrote the article. AB performed part of the laboratory procedures. CB, SC, MR, RP and AB critically reviewed the protocol, and then the manuscript. All the authors recruited patients, collected data and read and approved the final manuscript.

The Group for the Study of Involuntary Weight Loss (Grupul de Studiu al Scaderii Ponderale Involuntare, GSSPI): C Baicus, C Badea, E Balanescu, M Balea, S Caraiola, G Constantin, I Constantinescu, L Dimitriu, D Georgescu, O Ghizdavu, A Haidar, M Ghita, M Iacob, RA Ionescu, P Leru, A Marian, RB Mateescu, A Nanu, A Nicolau, I Nicolescu, D Nitescu, R Patrascu, V Pompilian, M Rimbas, S Tanaseanu, G Ticu, D Ursica, R Voiosu, Th Voiosu (Colentina Clinical Hospital), A Baicus (“I Cantacuzino” Institute).

## Pre-publication history

The pre-publication history for this paper can be accessed here:

http://www.biomedcentral.com/1471-230X/12/86/prepub

## Supplementary Material

Additional file 1 Table 4. Haematological parameters, adjusted for age, sex, inflammation and alanin aminotranspherase (as a surrogate for alcohol consumption) as predictors of colorectal/gastrointestinal cancer in patients with IWL – logistic regression. Click here for file

Additional file 2 **Figure 2.** The mean value of ferritin (mcg/L) in different locations of gastrointestinal tract cancers (colon = ascending, transverse and descending colon). Click here for file
