# Supplementary material for: Ferritin above 100 mcg/L could rule out colon cancer, but not gastric or rectal cancer in patients with involuntary weight loss
Source: BMC Gastroenterol. 2012 Jul 9;12:86. doi: 10.1186/1471-230X-12-86 (PMC3438089; doi:10.1186/1471-230X-12-86)
Supplement: Additional file 1 — Table 4. Haematological parameters, adjusted for age, sex, inflammation and alanin aminotranspherase (as a surrogate for alcohol consumption) as predictors of colorectal/gastrointestinal cancer in patients with IWL – logistic regression. [file 1471-230X-12-86-S1.pdf]

Table 4'. Haematological parameters, adjusted for age, sex, inflammation and alanin aminotranspherase (as a surrogate for alcohol consumption) as predictors of colorectal / gastrointestinal cancer in patients with IWL – logistic regression

	**Variable**	**Odds ratio**	**95% CI**	***P* value**
Colorectal cancer (14 patients)	sex (male)	2.3	0.34, 15.5	0.395
	age (years)	1.09	0.97, 1.23	0.155
	hemoglobin (g/dl)	1.2	0.7, 2.05	0.558
	MCV (femtoliters)	0.86	0.7, 1.07	0.176
	serum iron (mcg/dl)	0.99	0.95, 1.07	0.751
	RDW (%)	0.6	0.31, 1.18	0.142
	**ferritin<100 mcg/L**	**11.4**	**1.06, 121.2**	**0.044**
	ESR (mm/h)	1.02	0.99, 1.06	0.121
	CRP (mg/L)	0.99	0.96, 1.01	0.441
	ALAT (u/L)	1.0	0.93, 1.07	0.986
Gastrointestinal cancer (22 patients)	sex (male)	1.6	0.45, 5.62	0.475
	age (years)	1.04	0.98, 1.11	0.144
	hemoglobin (g/dl)	0.94	0.66, 1.33	0.731
	MCV (femtoliters)	0.96	0.86, 1.06	0.453
	serum iron (mcg/dl)	0.99	0.97, 1.01	0.523
	RDW (%)	1.02	0.74, 1.41	0.893
	**ferritin<100 mcg/L**	**7.00**	**1.6, 30.5**	**0.010**
	ESR (mm/h)	1.00	0.98, 1.03	0.655
	CRP (mg/L)	0.99	0.98, 1.01	0.622
	ALAT (u/L)	1.01	0.98, 1.04	0.332
