# Supplementary material for: Ferritin above 100 mcg/L could rule out colon cancer, but not gastric or rectal cancer in patients with involuntary weight loss
Source: BMC Gastroenterol. 2012 Jul 9;12:86. doi: 10.1186/1471-230X-12-86 (PMC3438089; doi:10.1186/1471-230X-12-86)

Figure 2. The mean value of ferritin (mcg/L) in different locations of gastrointestinal tract cancers (colon = ascending, transverse and descending colon)

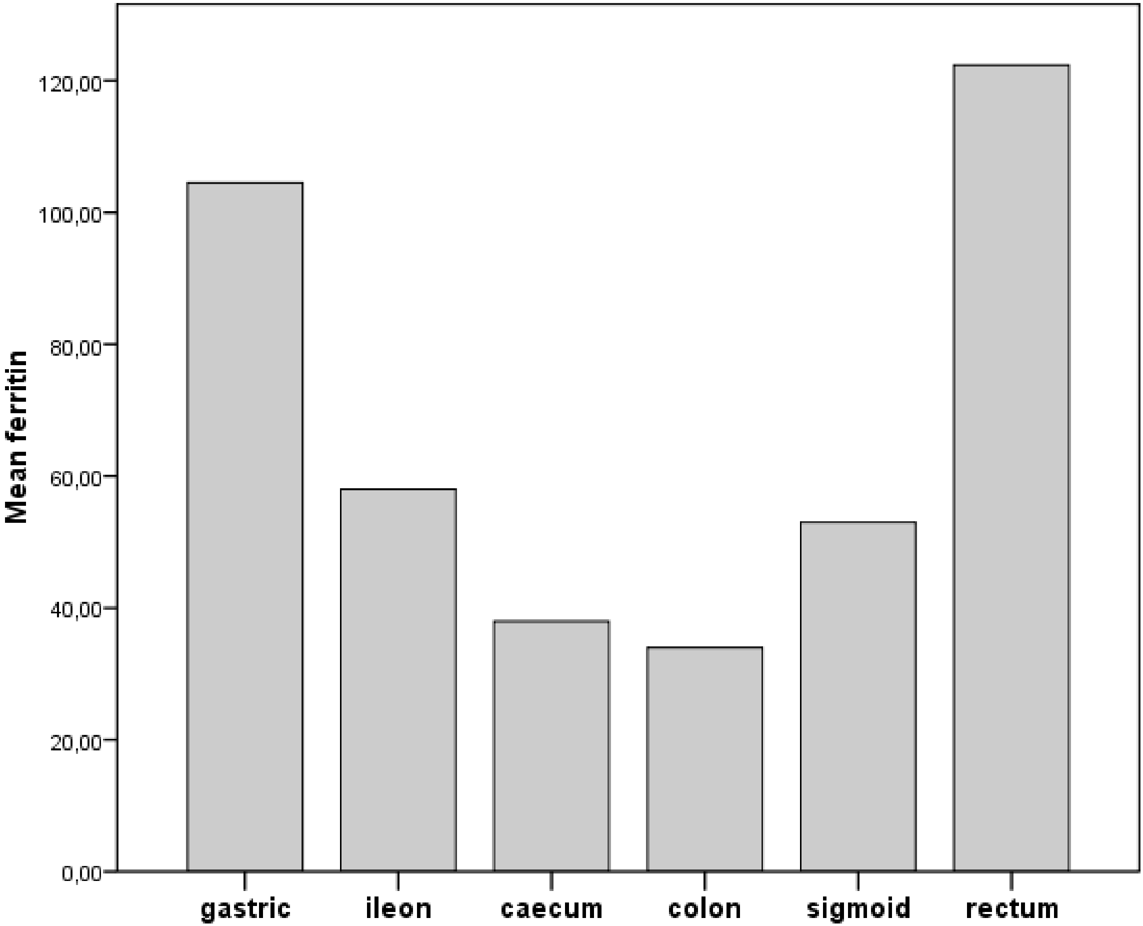

Supplement: Additional file 2 — Figure 2. The mean value of ferritin (mcg/L) in different locations of gastrointestinal tract cancers (colon = ascending, transverse and descending colon). [file 1471-230X-12-86-S2.pdf]
